# LeoA, B and C from Enterotoxigenic *Escherichia coli* (ETEC) Are Bacterial Dynamins

**DOI:** 10.1371/journal.pone.0107211

**Published:** 2014-09-09

**Authors:** Katharine A. Michie, Anders Boysen, Harry H. Low, Jakob Møller-Jensen, Jan Löwe

**Affiliations:** 1 MRC Laboratory of Molecular Biology, Structural Studies Division, Cambridge, United Kingdom; 2 Department of Biochemistry and Molecular Biology, University of Southern Denmark, Odense M, Denmark; University of Osnabrueck, Germany

## Abstract

*Escherichia coli* (ETEC) strain H10407 contains a GTPase virulence factor, LeoA, which is encoded on a pathogenicity island and has been shown to enhance toxin release, potentially through vesicle secretion. By sequence comparisons and X-ray structure determination we now identify LeoA as a bacterial dynamin-like protein (DLP). Proteins of the dynamin family remodel membranes and were once thought to be restricted to eukaryotes. In ETEC H10407 LeoA localises to the periplasm where it forms a punctate localisation pattern. Bioinformatic analyses of *leoA* and the two upstream genes *leoB* and *leoC* suggest that LeoA works in concert with a second dynamin-like protein, made up of LeoB and LeoC. Disruption of the *leoAB* genes leads to a reduction in secretion of periplasmic Tat-GFP and outer membrane OmpA. Our data suggest a role for LeoABC dynamin-like proteins in potentiating virulence through membrane vesicle associated toxin secretion.

## Introduction

Members of the dynamin family are large GTPases that couple nucleotide hydrolysis to membrane remodelling [1], [2]. The most studied dynamin family member (DFM) is the classical human dynamin 1, which, amongst a myriad of other functions, assembles onto invaginating membrane during endocytosis and forms large filamentous helical assemblies around the necks of budding vesicles. Dynamin 1 carries out a sequence of nucleotide-driven conformational changes that control its polymeric state and drive membrane fission [3]–[5]. In addition to classical dynamins, the protein family comprises dynamin-like proteins (DLPs), such as dynamin-related protein 1 (DRP1) and mitofusins, which are involved in mitochondrial fission and fusion, respectively [2]. Historically, DFMs have been found exclusively within eukaryotic cells but reports of dynamin-like proteins in bacteria show the family is larger than previously thought [6], [7]. The role of these proteins in bacteria is still largely unknown.

DFMs are activated by nucleotide-dependent dimerisation whereby two DFMs interact across the nucleotide-binding domains and both active sites contribute to hydrolysis, with the two GTP nucleotides occluded between the domains. Additionally, the dimers of DFMs also interact via their extended helical domains to form large polymeric structures assembling on lipid templates [8], [9].

Bacterial DLPs (BDLPs) share many of the qualities exhibited by eukaryotic DLPs—they polymerise, exhibit GTPase activity and assemble on lipid into filaments [6], [9]. Even though BDLP1 from *Nostoc* shares very low sequence homology with eukaryotic dynamins (less than 20% identity, with the highest similarity within the GTPase domain), the reported structures of human dynamin 1 [3], [5], [10] show essentially the same fold as BDLP1 [7] with three conserved structural units: the GTPase domain, the neck/bundle signalling element (BSE) and trunk/stalk domains [1]. Large structural differences observed between DFMs appear to be restricted to the angular relationship between these three core structural domains that are connected by flexible hinges, highlighting the many conformations that DFMs may undergo during assembly and during the hydrolysis cycle.

Further work on the bacterial DLP BDLP1 from *Nostoc* revealed how the protein interacts with the membrane lipid [9]. The hydrophobic paddle located at the BDLP1 ‘tip’ (at the extreme end of the trunk domain), inserts into the outer leaflet of the lipid bilayer promoting curvature. Classical dynamins appear to have replaced the paddle with lipid head group-specific Pleckstrin Homology (PH) domains that allow for topological and functional tuning within different cellular compartments. In terms of primary sequence between all DFMs, mitofusins such as Fzo1 [11] are most similar to BDLP1 and also share the same domain architecture with a hydrophic tip instead of a PH domain.

Here, we present data demonstrating that the *leoABC* genes, encoded within a pathogenicity island from enterotoxigenic *Escherichia coli* (ETEC) strain H10407, are dynamin-like proteins. This is important because LeoA has previously been linked with the secretion of heat labile enterotoxin (LT) through membrane vesicle (MV) biogenesis and release from the bacterial cell surface [12], [13]. Currently there are very few reports indicating functional roles of bacterial dynamins [14]. A role in membrane vesiculation would represent a conserved function for DFMs spanning evolutionary domains from prokaryotes to eukaryotes.

## Materials and Methods

### Cloning, over-expression and purification of LeoA for structural studies

The *leoA* gene (GenBank AAF22637.1) from *E. coli* ETEC strain H10407 was cloned into the T7 expression vector pHis17 (Bruno Miroux, MRC-LMB, personal communication), encoding the full-length protein with GSHHHHHH added at the C-terminus. Overexpression was achieved in *E. coli* C41 cells, growing in 2xTY media and induced with IPTG. Induction was initiated at an OD_600_ of 0.8 at a growth temperature of 34°C. Cells were harvested by centrifugation and stored snap frozen in liquid nitrogen at −80°C. Selenomethionine protein was over-expressed using a feedback protocol as described previously [15], [16]. Briefly, the same vector was transformed into C41 cells and a culture grown overnight at 30°C in 2xTY medium. Cell pellet from this culture was used to inoculate pre-warmed M9 minimal medium supplemented with glucose and MgSO_4_. 30 minutes prior to induction, amino acids, including selomethionine were added as solids to the culture and the growth temperature was dropped to 25°C. Induction with IPTG was initiated when OD_600_ was 0.8. Induction was carried out overnight and the cell pellet was harvested by centrifugation, snap frozen in liquid nitrogen and stored at −80°C. Cell pellets were lysed in 50 mM Tris, 300 mM NaCl, pH 8.0 with added DNase, in a Constant Systems cell disruptor at 25 kPSI. The cell lysate was centrifuged in a 45Ti ultracentrifuge rotor (Beckman) at 35 000 rpm. The supernatant was loaded onto two 5 ml HisTrap columns at 4°C (GE Healthcare). The column was washed with increasing steps of imidazole and eluted with 300 mM imidazole, pH 8.0. The proteins were gel filtrated using a Sephacryl S300 column (GE Healthcare) in 20 mM Tris, 100 mM NaCl, 1 mM EDTA, 1 mM sodium azide, pH 8.5. Fractions were pooled and concentrated to ∼20 mg/ml and stored at −80°C in small aliquots. Selenomethionine protein used for phasing was prepared under essentially identical conditions with the exception that either β-mercaptoethanol or TCEP was present in all buffers.

### Crystallisation and structure determination

1440 crystallisation conditions were screened in 100 nl sitting drops using a robotic facility and commercially available screens [17]. An initial hit was obtained using protein at 20 mg/ml in 300 mM ammonium sulphate, 10% PEG 4000, 100 mM sodium citrate, pH 5.6 (initial pH). This condition was optimised to 244 mM ammonium sulphate, 9.9% PEG 4000 and 100 mM sodium citrate pH 5.6, using 6 mg/ml of protein in an MRC Maxi (SWISSCI) sitting drop plate, using 500 nL of protein and 500 nl of reservoir solution. Wells were streak-seeded by hand with a cat's whisker using seed stock prepared from vortexed small crystals. Crystals were cryo-protected in 30% glycerol plus reservoir and flash-frozen in liquid nitrogen. Data was collected at beamline I02 at the Diamond Light Source, UK (Table 1). Crystals were indexed and integrated using MOSFLM [18] and further processed using the CCP4 package [19]. The structure was solved by multiple-wavelength anomalous diffraction (MAD) using the 3 Å data set and SHELXCDE [20] and Phaser [21] and built manually using MAIN [22]. Refinement was performed using REFMAC5 [23]. After initial building and refinement, molecular replacement was performed to solve the 2.7 Å native dataset, which was subsequently refined and rebuilt using REFMAC5 and MAIN. Final coordinates have been deposited in the Protein Data Bank with accession code 4AUR (Table 1).

**Table 1:** Crystallographic data.

Statistics	*LeoA SeMet*	*LeoA native*
Protein	full-length, C-terminal GSHHHHHH	full-length, C-terminal GSHHHHHH
GenBank ID	AAF22637.1	AAF22637.1
ATCC ID	35401	34501
**Data collection**		
Beamline	Diamond I02	Diamond I02
Wavelengths (Å)	0.9795, 0.9797, 0.9778	0.9795
**Crystal**		
Space group	C2	C2
Cell (Å)	185.1, 53.6, 73.9, 96.6°	185.5, 53.6, 73.9, 96.6°
**Scaling**		
Resolution (Å)	3.0	2.7
Completeness (%)^1^	96.6 (96.2)	97.8 (93.8)
Multiplicity^1^	7.2 (7.4)	3.3 (3.1)
ano completeness (%)^1^	99.1 (99.0)	
ano multiplicity^1^	3.7 (3.8)	
ano correlation^1^^,^ 2	0.594 (0.071)	
(I)/σ(I)^1^	18.5 (5.8)	8.2 (2.2)
R_merge_ 1	0.083 (0.317)	0.092 (0.416)
R_pim_ 1	0.050 (0.187)	0.087 (0.381)
**Phasing**		
Scatterer/mode	Se/MAD	
Number of sites	17	
Figure of merit	0.51	
**Refinement**		
R/R_free_ 3		0.216/0.289
Model		1–113, 121–571, 1 SO_4_, 50 H_2_O
Bond length rmsd (Å)		0.012
Bond angle rmsd (°)		1.47
Most favoured (%)^4^		93.0
Disallowed (%)^4^		0.6
MOLPROBITY score		97^th^ percentile
PDB ID		**4AUR**

SeMet data values for peak wavelength, only.

1 Values in parentheses refer to the highest recorded resolution shell.

2 Anomalous correlation coefficient between half sets (SCALA) [19].

3 5% of reflections were randomly selected before refinement.

4 Percentage of residues in the Ramachandran plot (PROCHECK) [19].

### Media, antibiotics, strains and plasmids for all other studies

Cells were grown in Lysogeny Broth (LB). When required, the media was supplemented with 30 µg/ml chloramphenicol, 30 µg/ml ampicillin and 1 mM IPTG. All strains used are derivatives of *E. coli* K12 and pathogenic *E. coli* H10407. Strains and plasmids are listed in Table S1 in File S1 and primers in Table S2 in File S1.

### Construction of strains

The *leoA*, *leoB* and *leoAB* knockout strains were made by replacing the 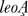 and *leoAB* genes with a chloramphenicol resistance cassette as described [24]. Briefly, a PCR amplification product generated using pKD3 as template and the primer pairs JMJ91+JMJ92, JMJ238+JMJ239 and JMJ238+JMJ92 was electroporated into *E. coli* H10407. Transformants were selected on LB agar plates containing 30 µg/ml chloramphenicol. Subsequently, the markerless strains (i.e. chloramphenicol sensitive) H10407Δ*leoA*, H10407Δ*leoB* and H10407Δ*leoAB* were made by flipping out the integrated antibiotic resistance cassette using pCP20. All constructs were verified by PCR analysis. The primers JMJ101+JMJ102 were used to verify H10407Δ*leoA* and the primers JMJ240+JMJ102 were used to verify H10407Δ*leoB* as well as, H10407Δ*leoAB*. The *leoA* 3xFLAG strain was constructed as described [25]. In summary, a PCR amplification product generated using pSUB11 as template and the primer pairs JMJ134+JMJ135 was electroporated into *E. coli* H10407. Transformants were selected on LB agar plates containing 30 µg/ml chloramphenicol. Subsequently, the markerless strain (i.e. kanamycin sensitive) H10407*leoA* 3xFLAG was made by flipping out the integrated antibiotic resistance cassette using pCP20. The primers JMJ101+JMJ102 were used to verify H10407*leoA* 3xFLAG. All constructs were verified by PCR analysis. IPTG inducible *leoA* plasmid pAB108 was derived from pNDM220 by digesting a PCR product with AatII and BamHI and subsequent ligation into pNDM220. The PCR product was generated by using the primers JMJ205 and JMJ206 on ETEC H10407 chromosomal DNA. The construct was verified by sequencing.

### Plasmids

To study the export of proteins into vesicles we have used a GFP-based reporter system developed previously [26]. Briefly, the full-length 5′-UTR and 129 bp of the N-terminal coding region of *torA* was amplified by PCR and subsequently fused to the 5′ terminus of GFP in the pXG-10 vector. This in turn generated the plasmids pAB107 (*torA′-gfp*). The relevant primer pairs are listed in Table S2 in File S1. Constructs were verified by sequencing using the primers pZE-CAT and pJVO-155.

### LeoA antibody

A polyclonal antibody was raised in rabbits against a solid-phase synthesised peptide chosen from the LeoA sequence ([C]-ELAEKSQAIRDNRQKLS-amide). The peptide was assessed by MADLI-TOF prior to use. Peptide and antibodies were produced by Cambridge Research Biochemicals (CRB, UK).

### Immuno-fluorescence microscopy

*E. coli* strain H10407 and AB109 were grown in LB medium at 37°C. At mid-exponential growth phase, 200 µl of cell culture were transferred to 1 ml of cold methanol and kept at −20°C for at least 60 min. Anti-FLAG antibodies were used at a 1∶100 dilution and Alexa488-conjugated goat anti-mouse IgG antibodies (Invitrogen) at a 1∶200 dilution. Cells were observed using a Leica DMRE microscope with a PL APO 100×/1.40 objective. Combined phase-contrast and fluorescence microscopic images were obtained with a Leica DC500 camera.

### Sub-cellular protein fractionation

LeoA protein from *E. coli* H10407, H10407Δ*leoA*, H10407Δ*leoB* and H10407leoA-3xFLAG was localised as described [27] with minor modifications. Briefly described, all strains were grown at 37°C in 50 ml of LB until OD_600_ = 0.6. The cells were harvested at 4000×g for 15 min at room temperature and subsequently re-suspended in 25% of initial volume in 20% sucrose, 1 mM EDTA, 20 mM Tris pH 7.6. The cells were stored at room temperature before harvest at 4000×g for 15 min at 4°C. Swelling of cells was induced at 4°C for 10 min by re-suspending the cells in 25% of starting volume in cold water. Finally the cells were harvested at 12,000×g for 15 min at 4°C and supernatant containing the periplasmic proteins was carefully isolated. Inner and outer membrane proteins as well as cytoplasmic proteins were isolated as described [28]. Sample volumes were concentrated using Amicon Ultra 3K devices (Millipore). Membrane vesicles were enriched as described previously [26].

### Vesicle GFP assay

The *E. coli* strains AB113, AB114, AB115, AB116 and AB 117 were grown at 37°C in LB until OD_600_ = 0.6. 200 ml cultures were harvested at 11,000×g for 10 min at 4°C. The supernatant was passed through a 0.22 µm sterile filter and subsequently concentrated using Amicon Ultra 10K devices (Millipore). Vesicles were isolated as described [26] and finally ethanol/acetone precipitated.

### Western blotting

Culture samples were grown under aerobic conditions to OD_600_ = 0.5. The cell pellets were re-suspended in SDS loading buffer (60 mM Tris-HCl pH 6.8, 2% SDS, 10% glycerol, 0.005% bromophenol blue, 5 mM EDTA, 0.1 M DTT) to a final concentration of 0.01 OD_600_ unit/µl and boiled for 5 min.

For detection of proteins a total of 0.05 OD_600_ unit of whole cell protein was loaded onto 4-12% Invitrogen NuPage (Novex) Bis-Tris mini gels. The gels were blotted for 60 min at 3.2 mA/cm^2^ in a Hoefer SemiPhor blotter tank (GE Healthcare) onto a PVDF membrane (Millipore) in transfer buffer (48 mM Tris pH 9, 20% methanol, 39 mM glycine, 0.0375% SDS). The α-GFP (Roche), α-GroEL (Sigma), α-FLAG (Sigma) monoclonal antibodies were diluted 1∶10.000, 1∶50.000 and 1∶10.000, respectively. The α-LeoA, α-TolC, α-Lep, α-OmpA, α-β-lactamase polyclonal antibodies were diluted 1∶1000, 1∶50, 1∶100 1∶3000 and 1∶3000, respectively.

Mouse and rabbit HRP conjugated secondary antibody was diluted 1∶2000 (Dako Cytomation). Blots were developed using Western Lightning Reagent (Perkin Elmer). The signal was detected and quantified using a ChemiDoc XRS station (BioRad).

## Results

### Bacterial dynamin-like proteins within the *tia* locus

The observation that LeoA is a large GTPase (64.2 kDa), with a putative involvement in membrane vesicle (MV) secretion [12], [13] prompted us to question whether LeoA could be an as yet unrecognised dynamin-like protein (DLP).

**Figure 1:**
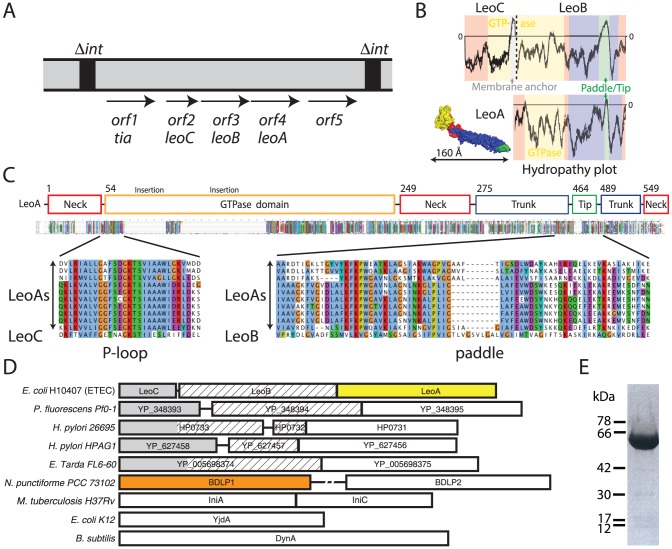
LeoA is part of a conserved putative operon. A: overview of the *tia* locus in *E. coli* ETEC H10407; modified from [13]. An annotated version of the locus with probable promoter and RBS sites is shown in Figure S1 in File S1. B: Surface plot of the LeoA monomer coloured by domains: yellow, GTPase domain; red, neck domain; blue, trunk domain; green, putative paddle region. Similarly coloured hydropathy plots (TMpred) provide transmembrane prediction for LeoA and LeoB, respectively. C: *Orf2* and *orf3* of the *tia* locus align well against *orf4*, which encodes LeoA and the alignment spans the entire length of LeoA. It seems that LeoA is encoded in tandem with another in sequence related *orf* that is split into two chains. *Orf2* and *orf3* have been renamed here *leoC* and *leoB*, respectively. Sequences aligned: LeoA (E. coli ETEC H10407), WP_011717023.1 (*Shewanella sp. ANA-3*), WP_007214706.1 (*Bacteroides cellulosilyticus*), WP_001006159.1 (*Helicobacter pylori*), WP_001006151.1 (*Helicobacter pylori*), WP_014535968.1 (*Helicobacter pylori*), WP_000787447.1 (*Helicobacter pylori*), WP_001006093.1 (*Helicobacter pylori*), WP_000787451.1 (*Helicobacter pylori*), WP_005966123.1 (*Fusobacterium periodonticum*). D: Tandem genes for bacterial DLPs are common. Previously reported were IniA and IniC, and DynA, which is a fusion of two DLP genes [37]. *Nostoc* BDLP1 occurs in tandem with BDLP2 [6]. YjdA does not seem to follow this pattern. LeoABC shows splitting of the first gene into two as shown in Figure 1B and C. Dimensions are approximate. A large non-coding region (525 bp) between BDLP1 and BDLP2 is indicated. E: Purified His_6_-tagged LeoA protein, over-expressed in *E. coli* and purified by metal affinity and size exclusion chromatography.

LeoA is encoded within the *tia* locus, which has been previously reported to comprise an island of five genes flanked by two integration sites [13] (Figure 1A and Figure S1 in File S1). Sequence alignments of LeoA with other dynamin family members (DFMs) including BDLP1 from *N. punctiforme*, YjdA from *E. coli*, and the eukaryotic mitofusin Fzo1 (while demonstrating low sequence identity of less than 20% Figure 1C and 1A), showed obvious conserved dynamin-like domains that include a predicted membrane-binding domain correctly positioned between the classical dynamin middle domain and GTPase effector domain (GED) (Figure 1B). The closest LeoA homologues are in other *E. coli* strains (H299 and TA007) and many *Helicobacter pylori* strains, which share approximately 30% identity (Figure 1C and legend).

Intriguingly two additional previously uncharacterised dynamin-like genes lie directly upstream of *leoA* (previously referred to as *orf2* and *orf3*) [13], and we have renamed these *leoC* and *leoB* respectively (Figure 1A–D). Since most bacterial dynamins identified to date are encoded in tandem [6] (with *E. coli yjdA* being a notable exception) this was not completely unexpected, however the arrangement of *leoC* and *leoB* is unusual in that a single, contiguous gene encoding a putative DLP has been split into two genes (Figure 1B, 1C and 1D). To be sure, we re-sequenced this chromosomal region from ETEC H10407 genome and confirmed the NCBI entry NC_017633.1 (Figure S1 in File S1). LeoC is 206 amino acids in length and encodes the N-terminus of a truncated GTPase domain that includes the P-loop and the Switch 1 GTP binding motif. The C-terminal 22 amino acids of this protein form a hydrophobic insert that is predicted to form an integral membrane anchor. LeoB is 572 amino acids long and constitutes the remainder of the DLP, providing a conserved Switch 2 GTP binding motif (consisting of the classical DFM consensus sequence DXXG) thus completing the GTPase domain, as well encoding (as per other DFMs) a predicted membrane binding domain between a middle domain and a GTPase effector domain (GED) (Figure 1B). The hydropathy plots of LeoC and LeoB end-to-end are reminiscent of that observed for BDLP1 [7] (Figure 1B). Taken together, LeoC and LeoB sequences appear to constitute a canonical DLP, supplemented by an additional GTPase domain membrane anchor that we note has also been observed in the ‘long’ isoform of the mitochondrial DLP Mgm1 [29]. The ‘split’ arrangement of the *leoBC* genes encoded upstream of *leoA* is conserved in a variety of pathogenic and non-pathogenic bacteria (Figure 1D), however the adjoining *tia* and downstream *orf5* genes are not conserved suggesting they have separate functional roles (Figure 1A).

### The crystal structure of LeoA shows it to be related to other dynamins

**Figure 2:**
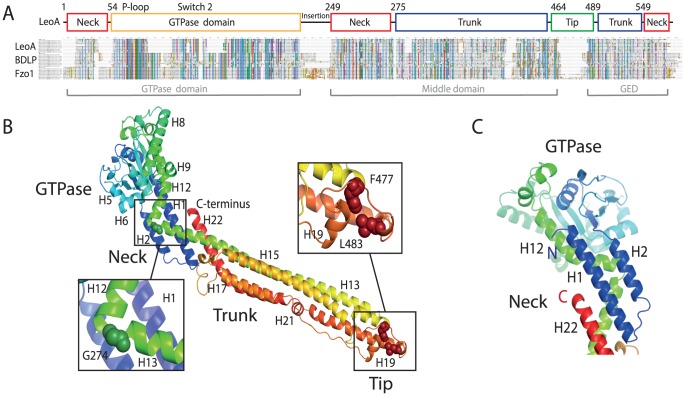
The crystal structure of LeoA. A: Schematic sequence alignment showing the similarity of LeoA to *Nostoc* BDLP1 and eukaryotic mitofusin Fzo1. In order to make the ClustalW alignment more stable, the 10 top hits from BLAST searches, with each of the three sequences, were included for each family. Conservation stretches across all three families, with all major domains of known function and fold being conserved, leading to the conclusion that LeoA is a *bona fide* bacterial dynamin-like protein. B: The 2.7 Å crystal structure of LeoA from *E. coli* ETEC H10407 shows an elongated molecule with the conserved GTPase domain followed by the trunk and tip regions. The conformation of the trunk relative to the GTPase domain, mediated by Gly274, is novel and reveals a ‘flattened’ conformation reminiscent of that observed in the DLP human guanylate-binding protein 1 [32]. The putative paddle region at the trunk tip is dominated by hydrophobic residues in conserved positions known to be critical for lipid binding in *Nostoc* BDLP1 [9]. A rainbow colour scheme is used from the N (blue) to the C (red) terminus. C: Close-up of neck and GTPase domains, rotated approximately by 180° with respect to main part of panel B.

In order to demonstrate that LeoA from *E. coli* H10407 is indeed a *bona fide* DLP, the protein was over-expressed and purified as a C-terminal hexahistidine-tagged fusion protein (Figure 1E). Monoclinic crystals of the protein in nucleotide-free state were obtained and the structure solved to 2.7 Å. (Table 1). This was achieved using seleno-methionine multiple-wavelength anomalous diffraction (MAD) with a 3 Å data set and then molecular replacement with the derivative structure leading to the final 2.7 Å model of the native protein. The structure (Figure 2B) was refined to R/Rfree values of 0.216/0.289 and the coordinates were deposited in the Protein Data Bank with accession code 4AUR (Table 1).

**Figure 3:**
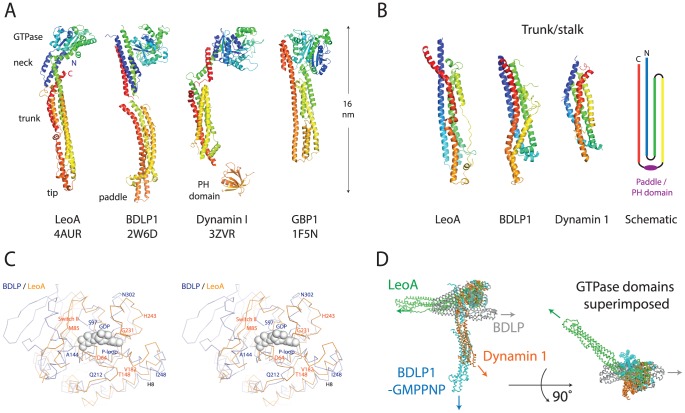
The structure of LeoA shows it to be related to prokaryotic and eukaryotic dynamin-like proteins. A: Comparison of different dynamin structures to the structure of LeoA, unequivocally showing it to belong to the dynamin family of proteins. B: Although the trunk structures superimpose poorly, their overall architecture and topology is conserved (scheme on the right). C: Stereo plot superposition of BDLP1 (PDB 2J68, GDP-bound form) and LeoA nucleotide-binding pockets. In this study GTP binding or hydrolysis by LeoA was not observed. The structure of the LeoA GTPase domain shows a distorted geometry, especially around loop and helices 225–248, although this sort of deviation is not uncommon for nucleotide-free structures of genuine nucleotide-binding proteins. BDLP1 is in blue, LeoA in orange, with the GDP from the BDLP1 structure in grey. The GTPase domains of BDLP1 and LeoA (residues 54–281 and 69–324, respectively) were aligned using Cα atoms only, with a resulting RMSD of 3.1 Å. D: LeoA reveals a novel conformation for the dynamin family. Superposition of BDLP1-apo (2J69), BDLP1-GMPPNP (2W6D) and human dynamin 1 (3ZVR) crystal structures is shown, using the LeoA (4AUR) GTPase domain as a reference. The attachment angle of the trunks to the GTPase domains is very different, and this presumably leads to different polymer assembly on lipid membranes and consequently different functional mechanisms.

The crystal structure of LeoA reveals a fold with all the hallmarks of a DLP including the key structural GTPase, neck/BSE and trunk/stalk domains (Figure 2ABC & 3AB) [5], [7], [10], [30]. Helix 1 and Helix 2 at the N-terminus form an extended helix-turn-helix motif as observed in BDLP1, that along with Helix 12 and Helix 22 at the C-terminus bundle to form the neck domain. Within the GTPase domain, which is an extended form of the canonical Ras GTPase domain (but smaller than that of BDLP1 by three beta strands, Figure 3C), Helix 8 is significantly longer than the equivalent region in human dynamin 1 but similar to that observed in BDLP1 (Figure 2B). In BDLP1, the N-terminus of this helix undergoes a substantial rotation as GDP is released from the binding pocket and may represent a mechanism for uncoupling dimerised GTPase domains. The LeoA trunk domain clearly shares the dynamin canonical fold as observed in other DFMs, and includes a putative hydrophobic membrane binding/paddle domain (Figure 2B and 3B). Flexibility between the neck and trunk domains has been inferred from comparisons of the crystal structure of BDLP1 and electron microscopy reconstructions of BDLP1 assembled on lipid [9]. Consistent with this, the trunk of LeoA is oriented orthogonal to Helix 12 in the neck, a conformation not observed previously. This 90° kink between trunk Helix 13 and neck Helix 12 is mediated by Gly274 (Figure 2B, inset) and represents the equivalent of Hinge 1a in BDLP1 [9]. Superposition of the LeoA and BDLP1 GTPase domain apo-forms show the LeoA trunk domain to be angled in the opposite direction to the BDLP1 trunk (Figure 3D). These differences in a single inter-domain angle should not distract from the fact, though, that the fold of all domains is conserved, making LeoA a clear member of the dynamin family.

At the tip of the trunk domain, the predicted paddle region of LeoA is shorter in length than the corresponding region observed for BDLP1, however the positions of hydrophobic residues known to be critical for membrane binding in BDLP1 [9] are similarly conserved (Figure 2B). Crystal packing analysis did not reveal any obvious physiologically relevant interactions of LeoA monomers with each other.

### LeoA localises to the periplasm

**Figure 4:**
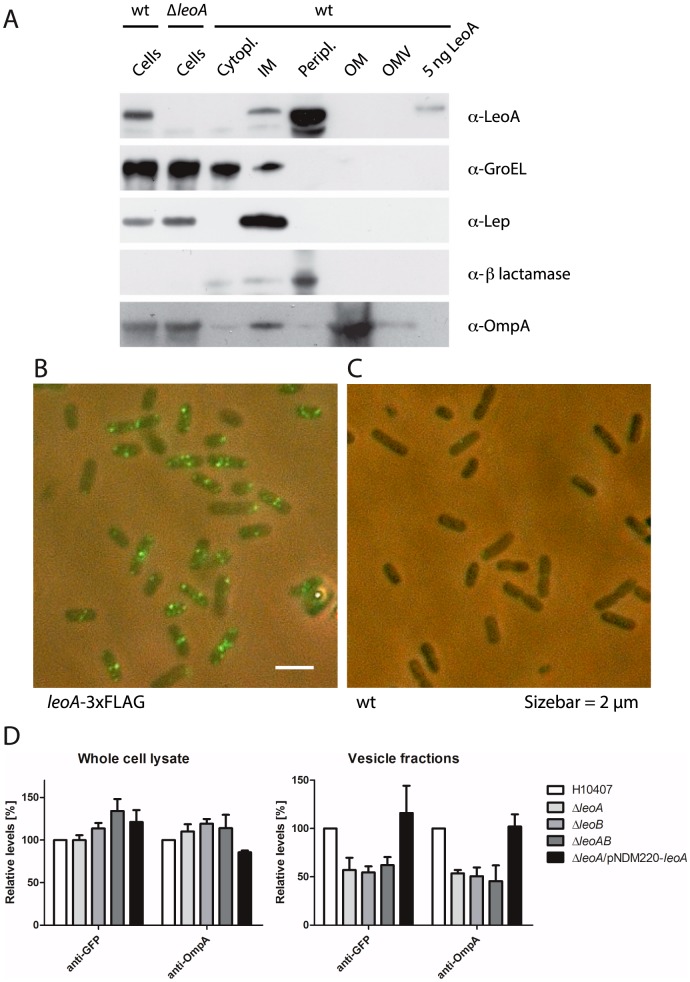
LeoA is localised in the periplasm and the *leoAB* genes enhance vesicle-based protein export. A: Using a polyclonal LeoA antibody, the sub-cellular localisation of LeoA in WT *E. coli* ETEC H10407 was investigated. The protein at endogenous expression levels is easily detected and this signal disappears in a *leoA* KO strain, demonstrating specificity. A classical fractionation experiment with sub-cellular marker proteins clearly shows LeoA to be localised in the periplasm and possibly in the inner membrane. Figure S5 in File S1, top shows the same experiment with a 3xFLAG-tagged version of LeoA. B: Immunofluorescence using the *leoA*-3xFLAG fusion strain shows a punctate pattern, which is specific to the presence of the fusion (C, right). See Figure S2 in File S1 for quantification of preferential polar and midcell localisation. D: Quantified Western blots demonstrating the influence of Leo proteins on protein secretion into the culture supernatant, presumably via vesicles. Both OmpA and a twin arginine-exported GFP reporter construct tend to accumulate in H10407Δ*leoAB* whole-cell lysates (left). GroEL served as an internal loading control (Figure S6 in File S1 shows the original blots and quantification data from two biological replicates). Right: conversely, OmpA and Tat-GFP levels are reduced by about 50% in culture supernatants (vesicle fractions) from the H10407Δ*leoAB* mutant strain. Supplying extra LeoA protein from a plasmid reverses the effect of the *leoA* deletion (last column).

Mass spectrometry analysis revealed that a small amount of OmpA and OmpX, two highly abundant, beta-barrel, integral outer membrane proteins, were co-purified with recombinantly expressed LeoA (not shown). This is consistent with the previous findings by Brown *et al*. [12], who reported direct interaction between LeoA and OmpA as well as greatly reduced OmpX levels in a *leoA* deletion mutant. The interaction of LeoA with proteins in the outer membrane suggests that it is exported from the cytoplasmic space despite there being no obvious targeting sequence. In order to examine the expression and subcellular localisation of LeoA we used polyclonal antibodies raised against LeoA-His_6_ for immunoblotting. The Western blot in Figure 4A shows that the antiserum recognised purified LeoA protein and that H10407 cells grown to mid-exponential phase expressed LeoA, whereas the protein was absent in an isogenic *leoA* deletion mutant. Subcellular fractionation demonstrated that LeoA was found primarily in the periplasmic compartment and to a lesser extent associated with inner membrane. The compartmental control proteins, GroEL, leader peptidase protein (Lep), β-lactamase and OmpA were found to localise to the cytoplasm, inner membrane, periplasm and outer membrane, respectively, thus validating the biochemical fractionation protocol used. Similar results were obtained with a 3xFLAG-tagged version of LeoA (Figure S5 in File S1, top) and periplasmic localisation is also not affected by deleting *leoB* (Figure S5 in File S1, bottom). Interestingly however, there was no sign of an interaction of recombinant, untagged LeoA *in vitro* with lipids, synthetic or natural, from either a bacterial or eukaryotic source, and under a wide range of conditions, including in the presence of various metal ions, crowding agents, and with and without nucleotides (not shown, see Discussion).

As the anti-LeoA serum was of insufficient titer to allow immunofluorescence microscopy, we constructed a chromosomal fusion allele in which the LeoA C-terminus was fused in frame to a triple-FLAG tag [25]. Monoclonal anti-FLAG antibodies were used to detect LeoA-3xFLAG fusion protein expressed from its normal genetic context. As shown in Figure 4B, LeoA-3xFLAG forms distinct punctate foci, the number and exact position of which varied amongst individual cells, although a preference for the poles (31.2% of foci observed, n = 250 cells) and mid cell (46.4% of foci observed, n = 250 cells) was noted (Figure S2 in File S1). Wild type H10407 cells did not display fluorescent staining confirming antibody specificity (Figure 3C).

### The *leo* genes are important for vesicle release

Most of the heat labile enterotoxin (LT) released by ETEC strains was found in association with membrane vesicles (MVs) [31]. The finding that LeoA was required for maximal secretion of LT from the ETEC strain H10407 and resulting fluid accumulation in a rabbit ileal loop model [13] points to a function of LeoA in MV release. Indeed, a higher number of vesicles for wild-type H10407 compared to the isogenic *leoA* deletion strain were observed by electron microscopy [12]. In order to quantitatively assess the importance of *leo* genes in the production of MVs we constructed a GFP-based reporter system in which a twin-arginine export signal was fused to *gfp*. This gene fusion allowed for detection of GFP in purified vesicle fractions by fluorescence or Western blotting, in addition to monitoring levels of outer membrane protein OmpA. Vesicles were purified from wild type H10407 and isogenic *leoA*, *leoB*, and *leoAB* deletion mutants. Using culture optical density as a normalisation parameter, protein levels were measured in whole cell lysates and vesicle fractions by quantitative Western blotting (Figure 4D). H10407, H10407Δ*leoA*, H10407Δ*leoB* and H10407Δ*leoAB* strains display modest but similar amounts of Tat-GFP and OmpA accumulation in whole-cell lysates (Figure 4D, left, Figure S6 in File S1). In contrast, Δ*leoA*, Δ*leoB* and Δ*leoAB* strains contain approximately 50% of normal amounts of both Tat-GFP and OmpA protein in the purified vesicle fractions (Figure 4D, right, Figure S6 in File S1). Tat-GFP and OmpA levels in vesicles could be restored by expressing LeoA from a plasmid. Overall, deletion effects do not seem to be accumulative, suggesting the *leo* genes act in concert.

## Discussion

LeoA from the pathogenic *E. coli* ETEC strain H10407 had previously been reported as having homology to bacterial and eukaryotic GTPases [12]. Here, we show by sequence analysis and X-ray crystallography that LeoA is a bacterial dynamin-like protein (DLP). This observation is of particular interest as LeoA has previously been implicated in the release of LT toxin via membrane vesicle (MV) secretion from the cell surface [12], [13]. These results provide a tantalising glimpse into the potential role of DLPs in bacteria.

Whilst the crystal structure of LeoA reveals the canonical structural domains of other DLPs, the conformation of the trunk relative to the GTPase domain and neck is novel and is another example of the increasing number of conformational variants observed for DFMs in crystal structures. After superimposing the GTPase domain of LeoA upon that of BDLP1 (apo and GDP-bound) and human dynamin 1 (apo), the LeoA trunk is observed to be fully extended, pointing in an opposing direction to the nucleotide binding pocket of the GTPase domain, and yields a ‘flattened’ conformation that is more reminiscent of human guanylate-binding protein 1 (hGBP1) [32]. Similar conformational flexibility at the interface between the BSE and stalk in human mitochondrial Drp1 has been reported [33]. Thus, unlike the nucleotide-free and GDP-bound form of BDLP1 (PDB 2J69 and 2J68) [7], LeoA in the apo form is extended, and nucleotide and/or lipid binding is not required to adopt this conformation. Note that the DLP hGBP1 also has its helical C-terminus angled in a conformation reminiscent to that observed in LeoA [32].

Our bioinformatic analysis suggests that the two genes (*leoC* and *leoB*) immediately upstream of *leoA*, (and likely to be co-expressed with LeoA) are also DLPs. The observation of two genes (*leoC* and *leoB*) that together should make up a functional DLP is novel. We can only speculate why the protein has been split into two parts but such an arrangement may be important for regulating assembly, transport or nucleotide hydrolysis.

Many bacterial genomes encode more than one DLP, which are often encoded back-to-back, or in some cases fused together as for *Bacillus subtilis* DynA [6]. The general tandem encoding of bacterial DLPs suggests some form of cooperation between the two proteins [34]. Since dynamin-like proteins form stalk and GTPase-domain dimers that associate to polymerise into large structures, it seems likely that the two dynamin-like proteins always occurring in a single operon in bacteria may generally form heterodimers. If such heterodimers form, it poses the interesting question as to whether they form across the GTPase domains and are required for GTPase activity (or indeed GTP binding), or if they interact between homodimers to form larger hetero-oligomers via the helical domains, or both. We were unable to observe any nucleotide binding or hydrolysis for LeoA alone, under a wide variety of conditions including in the presence of both cations and lipid (Figure S3 in File S1). Although this contradicts previous findings [12], it is consistent with the nucleotide-binding pocket of the GTPase domain being occluded in the LeoA crystal structure (Figure 3C). Given most of the residues responsible for hydrolysing GTP are conserved, the observed lack of nucleotide binding might be explained if LeoA forms a heterodimer with chimeric LeoBC across the GTPase domains, and hydrolysis/GTP binding requires components from both LeoA and LeoBC.

We found that *leoB*, and *leoA* (similar to previous reports) have a likely role in mediating membrane vesicle (MV) secretion. Individual gene knockouts of *leoA* or *leoB*, and *leoAB* combined induced a roughly 50% decrease in MV release as assayed using a Tat-GFP reporter system and by measuring OmpA levels. This effect is not caused by obvious changes in growth or morphology (Figure S4 in File S1). It therefore seems likely that the LeoA and LeoBC proteins have roles in MV secretion in ETEC strain H10407. In this context it is interesting to note that the putative DLP IniA from *Mycobacterium tuberculosis* has also been functionally implicated in bacterial secretion. IniA has been shown to be important for the export of the anti-tuberculosis drugs isoniazid and ethambutol, and it was concluded that it might be operating as an efflux pump [35]. In light of the results presented here, it is possible that IniA functions to secrete isoniazid and ethambutol indirectly within MVs. Mycobacteria have been shown to secrete toxic or immunomodulatory molecules within MVs comprised of polar lipid material probably garnered from the plasma membrane. Thus, IniA would need to assemble on the periplasmic side of the plasma membrane to bud a vesicle (comprised of plasma membrane and the mycolic acid ‘outer membrane’) out of the cell.

Our *in vivo* biochemical fractionation assays revealed LeoA resides predominantly within the periplasm in *E. coli* H10407, and we also observed LeoA binding to outer membrane proteins as previously reported [12]. However, it is unexpected to find LeoA in the periplasm, given the presence of the GTPase domain and the fact that nucleotides are absent from the periplasm. We also note that none of the Leo proteins contain an obvious signal sequence for periplasmic export despite the periplasmic localisation data being consistent with a role in MV secretion. The mechanism of membrane curvature exerted by dynamin proteins causes membrane distortion localised to the side of the membrane that the DFM binds. Consequently, to bud a vesicle out of a cell, DLPs would be required to be on the periplasmic side of the inner membrane, or on the outside of the cell altogether. Indeed, there are reports of bacteria secreting vesicles comprised of both inner and outer membrane [36]. Clearly, alternative models are possible, for example involving vesicles in the periplasm filled with LeoABC and these findings require further investigation.

We were unable to observe LeoA alone binding bacterial, mammalian or synthetic lipids *in vitro* despite the paddle domain of LeoA showing conservation of hydrophobic residues that interact with lipid in the case of BDLP1. The LeoA paddle is also glycine rich making it likely that this region of the protein would exhibit flexibility if presented with a hydrophobic environment. It seems likely this paddle region might comprise a membrane-interaction domain, consistent with other members of the dynamin family. However, lipid interaction may depend on or be modulated by LeoBC interactions. Similar data demonstrating variable lipid binding of DFM proteins has been reported for DynA from *B. subtilis*, which comprises two entire DLPs (DynA_D1_ and DynA_D2_) fused together. Here, only DynA_D1_ was shown to interact with the membrane whilst DynA_D2_ showed no affinity. Also, DynA-mediated membrane fusion *in vitro* was dependent only on magnesium and not GTP [6].

The presence of LeoA within the bacterial periplasm and its potential requirement for hetero-dimerisation with LeoBC for activation is reminiscent of the eukaryotic Mgm1 family of DLPs [29]. Mgm1 exists as two isoforms, long and short, in the mitochondrial intermembrane space, which is topologically equivalent to the bacterial periplasm. The long isoform has an N-terminal membrane anchor similar to that observed in LeoC and means that the GTPase domain is maintained in close proximity to the membrane surface. The short isoform is not constitutively bound to the membrane and is soluble, as with LeoA. Neither long-Mgm1 nor short-Mgm1 exhibit GTPase activity alone, but exhibit complex activation dynamics depending on hetero-dimerisation and addition of lipid [29]. Given that the predicted trans-membrane helix of LeoC, unless cleaved, would tether the GTPase domain of any LeoBC complex to the membrane surface, it is interesting to speculate that the novel ‘flattened’ conformation of LeoA could be an adaptation to allow both GTPase dimerisation and lipid binding whilst lying flat against the membrane.

Indeed, since the majority of bacterial dynamin-like proteins are encoded within putative tandem operons as previously discussed, and in light of our biochemical studies it is probable that hetero-dimerisation is central to the molecular mechanism of this class of DLPs. We attempted to test this hypothesis directly but were unable to obtain either individually, in combination, or expressed as a chimeric fusion, LeoB and LeoC in sufficient quantities for biochemical studies. This remains an urgent focus for future studies and may well require the study of related systems.

## Supporting Information

File S1 (PDF)
