# Supplementary material for: LeoA, B and C from Enterotoxigenic Escherichia coli (ETEC) Are Bacterial Dynamins
Source: PLoS One. 2014 Sep 9;9(9):e107211. doi: 10.1371/journal.pone.0107211 (PMC4159319; doi:10.1371/journal.pone.0107211)
Supplement: File S1 — (PDF) [file pone.0107211.s001.pdf]

Supporting Information File (S1)

LeoA, B and C from enterotoxigenic *Escherichia coli* (ETEC) are bacterial dynamins

Katharine A Michie, Anders Boysen, Harry H. Low,
Jakob Møller-Jensen and Jan Löwe

Supporting Methods

Equilibrium Dialysis

70 µl LeoA-His_6 (180, 50, 10, 5, 1 and 0.5 µM) in 25 mM Tris, 1 mM EDTA, 1 mM NaN_3, 300 mM NaCl, pH 8.5 was dialysed in DispoEquilibrium dialysis cassettes (MWCO 10 kDa, Harvard Apparatus Holliston MA) against 100 nM [α-32P] GTP in 25 mM Tris, 1 mM EDTA, 1 mM NaN_3, 300 mM NaCl, 200 nM $MgCl_2$, pH 8.5. Samples were left to equilibrate at room temperature for 42 hrs. Blank samples without protein were used to assess when equilibrium had been achieved. 10 µl aliquots in triplicate of each chamber were added to scintillant and counted on a Beckman LS 6000 Series liquid scintillation system. DPM were calculated from 10 minute counts of each sample.

Filter Binding Assay

LeoA-His_6 (100 and 200 µM) was incubated with 0.9 mM [α-32P] GTP in working buffer (25 mM Tris, 1 mM EDTA, 1 mM NaN_3, 150 mM NaCl, 1.8 mM $MgCl_2$, pH 8.5) for 10 minutes at 4˚C. Triplicate 10 µl aliquots of each reaction were rapidly filtered by vacuum through nitrocellulose membrane that had been pre-equilibrated in cold working buffer. The filter was washed with 100 µl of cold working buffer three times, and the filter was added to scintillant and counted on a Beckman LS 6000 Series liquid scintillation system. Disintegrations per minute (DPM) were calculated from 10 minute counts of each sample and used to determine the number of nanomoles of GTP bound to the protein sample. *Methanococcus jannaschii* FtsZ (26 and 13 µM) was used as a positive control. Solutions of working buffer and GTP only were used to determine background retention of the radio-labelled nucleotide.

GTPase assay

GTP hydrolysis was assessed using a PiColorlock ALS assay from Innova Biosciences, Cambridge as described by the manufacturers instructions. LeoA-His_6 at 2.5 µM (in 25 mM Tris, 150 mM NaCl, 1 mM EDTA, 1 mM NaN_3, 2 mM $MgCl_2$ was incubated at room temperature with 1 mM GTP. Samples were assayed at 5, 10, 20, 30, 40 and 50 minutes, by withdrawing 50 µl of the reaction and adding 200 µl of the ALS mix and incubating on ice. Absorbance at 632 nm was used to determine the free phosphate ions released. A phosphate standard curve was prepared using sodium

phosphate. The reaction was normalised including a control without LeoA-His_6.

Figure S1. Annotated putative *leoABC* operon.

```
4227387   TTGTCGATCGATTCGATCGAATTTTCTCTTTTTACCCTGTCTTTTGCATG
          GTTTTAAAATAAGCAACCTTCTTTGTCGGGTAGGGTAAGAACCAACGGAG
          ATGATTGAAATGAAAAAGGTTATTGCGGTTTCAGCGCTTGCAATGGCAGG
          TATGTTTTCGGCCCAGGCTCTGGCTGATGAGAGCAAAACAGGCTTTTATG
          TGACCGGTAAAGCCGGTGCTTCAGTTGTGATGCAGACTGACCAGCGCTTC
          CGTCAGGACTTTGGGGATGATGTTTATAAGTATAAGGGCGGTGATAAAAA
          CGATACTGTATTTGGTGCCGGCCTTGCAGTGGGCTATGATTTTTATCAAC
          ATTACAATGTTCCAGTACGCACGGAAGTGGAATTCTATGGCCGTGGAGCT
          GCAGACTCCCGTTATACACTGGATACATGGCGTTCTCCGATGGGGGATGG
          TGGTCGGGAAGACACACAAAATAGGCTCAGTGTGAATACCCTGATGGTGA   tia
4226887   ACACGTATTATGATTTCAGAAACAGCAGTGCATTTACTCCATGGGTATCT
          GTTGGCCTGGGTTATGCACGGGTACATCATAAGGCGACATATATTGATAC
          CTCCTGGAATGAATCTGGCGAGATAAGTGATATTTCTGCGTTACATTACT
          CGGGATATGATAACAACTTCGCATGGAGCATTGGGGCCGGTGTTCGCTAT
          GACGTAACCCCGGATATCGCTCTTGACCTCAGCTATCGCTATCTGGATGC
          TGGTAAATCCTCCCTGTCTTACAAGGATACAGAAGGGGATAAATATAAAT
          CAGAGGCTGACGTTAAAAGTCATGACATTATGCTTGGGGTAACTTATCAT
          TTCTGATGAGAATGTTTATCCATGCCTGCATATTGCAAATGCAGGTGTGG
          GTATTATCCACAAATAAAAAGGAAAGTCTGGATGGTATATCCTCGTCCGC
          CAATGACTTTTATTTGCACGAAATGTAACTGGAAAAAAACAATTACCCAT
4226387   ATTAACTGTATAAGATCTTTTTCTTCATGCCCCCGATGTGGTTGTAAAGA
          AATTGCAGTACGTCGTGCAGGTATCATCCCGATTTTATTGGCAGCATTTA
          CGTCAGGTTCTTTTCCTGAACGTTGATATTGTGATTTTCATTTTTCGGTA   promoter?
          ATATTTCTGTCTATTATTGTTGAATAATAACTGGATATATAGAAGAAGCG
          AGGTCCTTATGGATGTGAACAGGGCACAATGTATAACGACGAAAGAATAT
          TTTAGCCGTCTGTACGATGATATTTGTCATAATCTTCAACAGACAACAGA
          TGATATTTCAAAACTGCATGTTGATAATGAAGGCGGAAAAAAACAGCTTA
          ACGTAATGATGGAGCAACTGCAGACATTACAGAATAATTTTAATCATAAG
          CTAAATTATCTGAAACAGCATGCTGAATGGGACAGATTTACTGTTGCCTT
          TTTTGGTGAAACAAATGCTGGTAAAAGTACCATTATTGAATCTTTAAGAA   leoC
4225887   TTTTTTTTGATGAACTCTCTCGCAAACAGCTTTTACAAAATAATCAGAAT
          GATTTGCAACAGGCGGAGCAGGTGTTGTGCGAAAACCTGGAAATGCTACG
          TCGTGATTTAATACAGGCATATAGCGAAGTTGCCAATAAAACTCGTGACA
          TCAGATTGTCTGCTAAGTGTTTGCAACAAATTATCGCTAATGAAAGTCAG
          TCTCGTTTGCAAATTTTACAACAGCAAACTCATGCTAAAGTTCGTTTCAC
          GTTATTAGCGACTGCGTGTGGCTGTTTTATAGCAGGTGCAGGCGGGATGG
          CAGCCTTGTTGAGTCAAATATGGTAGGGGGCTGACAATGAGTGATACATG   RBS
          GTCTTCTGTAGAGGAGAATCCGGAGCGGGTATTCAGGGATACTCACCCGG
          AAGGTAACCCTGCGAGTGATATTCTGTACTTACTGGAGCAAAACTGGTCG
          CGCTTTGTCAGTTCTTTTATTCTGACAGAGAGATTAAAAAACAGGGCAAG
4225387   GCGCGTTGATGGTTTAATCATTGGTACTGGTGAATCCGATTTCACTAAAG
          GTAATACCCATTATGATTTATGTATTGACGATAAGGTTTTCCGGTTAATT
          GATGTACCTGGCATTGAAGGCAATGAAAATTGCTATGCTGATCTGGTTAA
          AGAAGCTGTTGCGCAAGCGCACCTGGTGGTTTATGTCAACGGTACTAATA
          AAAAACCCGAAGCAACAACAGCCAGGAAGATCAAATCTTATCTGGAATAT
          GGTACTCAGGTTTATCCGTTGATCAATGTGCGCGGTTATTCTGAGGCCTA
          TGAGTTTGATGAGGATCGTATCGATCTCGAACAACAGGGTAGGGCTGGTG
          ACGCTTTATGTCAGACTGTTGGGGTTCTCGCTTCCGTATTAGGGCCGGAT
          GTACTGCTGAATGGGAACTATGTGCAGGGATTGCTGGCGTTTTCGGCGTT   leoB
          GGCTTATGACGACAATACACAAAGCACGACCATTCATCCTTCACGTGATC
4224887   TTAATCTGGCGGTATATCAACGCGGTTTTTTTGATGTATTTCCTTCTCGT
          CAGGAAATGCTGACATTTAGCCAGATTGATGCCGTCGCTCAGACAATTAA
          AAATAAGGTCGCAACGTTTCGTGAGGATATTATCGAAAGCAATAAAGGTA
          AAGTCCGTGAAACGCTGGAGCAATACTTACAGGTATTTGAAGAGCAATTG
          AGCAGCCATCACTGCTTTTTGCAAAAGATAATGCCGGAATTTGAAAAATG
          TCGGGTTGCATTCAGAAATGCGACTGGTGAATTTGAACGCCGTATCGCGA
          ACAGTCGTCGTAATCGCTGGAATACCCTGTTTAATGACCTGAGCAATGCC
          AGTGACGATATTGTCGAAGATAATTTTGGTGATAACAGTGCTATTTCCAC
          ACGGATCCGTCGTGAGTATGAAAAGCGCCGTGAAATTATGGAACAAGTGA
          TGCTGGAGGATACCGAAGAGAGTGTAAAGGTATTACAGCAGCAAACACTT
4224387   CAGGCGGTAAATCGTCTGCTGGAAGATATTCACAATGTGGAACTTCAACA
          CCCCCTCAGTTTTGCTCACAGCCACAGTATTGAGTTTAGTGGTGAGATGG
          TACCCCGTTACGATCTGGGGGTGGCTGATTTTAGTTCAATGGTTTTAAAA
```

GTTGGCAGCTATGCCATGTCAGGCAGTGCGATTGGCAGCATCTTTCCCGT
TATTGGTACGCTCGTTGGCTCTGTGCTTGGCGCGCTGGTCGGCATAATAA
TGACCGTTGCAGGTATTTTTACCAGTAAAGCGTCAAAAATACGCAAAGCA
CAAGGAAAAGTACGCGACAGGCTGGAAGAGGCGCGGGATAAATCTCTCGA *leoB*
CAGCGTTGCCGATGAAACCCGCCTCCTGGTTGCTGCTATTGAAAAAGAAC
TTGAGAGTGGGCTGCTGCAAAAAGTCAACGATATGCAGGTGGCTCTGCAA
CAGTCTGTTGCCATTTTCGAAACACAAATCAGTCAAATCACAACATTAAA
4223887 AAAACAACTGG**AGGGCA**TGCCTTATGGAACAATTCAAACAGTTCAGTATT **RBS**
GAAAAACAGGCTGCGATTAACTCGCTATTACAGTTGCGCGGAATGTTAGA
AATGCTGGGAGAGATGGGGATAAACATCAGCGACGATTTACAAAAAGTCA
CTTCTGCAATTAATGCCATCGAATCTGATGTCCTGCGTATTGCTCTGTTG
GGGGCGTTCTCCGATGGCAAAACCAGCGTTATCGCCGCATGGCTGGGTAA
AGTAATGGATGATATGAATATTTCCATGGATGAGTCCTCCGATCGGTTGA
GTATTTACAAACCGGAAGGTCTGCCAGATCAGTGTGAAATTGTTGATACG
CCCGGGCTGTTTGGTGATAAAGAGCGCGAGGTGGACGGGAGACTGGTGAT
GTATGAAGACCTGACCAGACGCTATATATCTGAAGCACACCTGATTTTTT
ACGTGGTTGATGCCACGAACCCGCTCAAGGAGAGCCACAGCGACATCGTA
4223387 AAATGGGTATTGCGCGATTTGAATAAACTTTCTTCAACCATCTTTGTTAT
CAATAAAATGGATGAAGTGACCAGCCTGACAGATCAGGCACTGTTTGACG
AGCAGGCGGCAATTAAAAAGGCGAATCTGAAAGGCAAATTGCAACGTGCA
GCGGATCTTACCGCTCAGGAGTGTGAGCAGCTAAATATCGTCTGCGTCGC
CTCAAACCCAAATGGTCGCGGCTTAACGTACTGGTTTACTAAACCCGAAC
ATTACGAAAGTCGTTCGCGCATTAACGATCTTAAAAATGCTGCCACCGAA *leoA*
ATACTGAAAACTAATGTGCCAGAGGTCTTGTTGGTGAAAACAGGTATGGA
CGTGGTGAAAGACATTGTCATTCAGCGTGTCACCCTGGCCAGCAGACACC
TCGATGAACTGAATACCTTTGTCGAGAAGAATGACGAGGATATGCACCGT
TTTAGTAACGACATAAAGCAAAGCCGCATTGAGGTTAAACGGCTGGCTGG
4222887 AGAATTATTTGAAGAGCTGAACCTGATGGAAAAACAGCTGATGAGCCAAC
TGCGTCCACTCGATCTGGACGATATCCGCCCGTTTATGGACGATGAGTTG
GGATATACAGAAGATGGGGTTGGCTTTAAGCTTCACCTGCGCATTAAGCA
GTCAGTTGATCGCTTTTTCGAACAGTCGACAGCGGTCTCGCAACGCTTAT
CTGATGATATTACTCGCCAGTTGAGCTCCAGCGAGAGCTTTCTAAGTGGC
TTAGGCGAGGGGGCGTTTCGTTCTTTAGGCGGGGCCTTTAAGGGGGTGTC
GAAAATTAGTCCGGCGACGCTCAAAACGACAATTTTGGCCGCACGCGATA
CGATTGGCAAACTAACGGGATATGTCTATAAATTTAAACCCTGGGAGGCC
ACCAAACTGGCCGGGAGCATTGCCAAATGGGCAGGTCCTGTGGGTGCAGC
GTTTACGATTGGTTCTGATCTATGGGATGCCTATAAAGCACACGAGCGGG
4222387 AGCAGGAACTGAAAGAAGTAAAAGCCTCGCTGGCGAAAATCATTAAAGAG
CCGTTTGAAGATATCTATGACGTGCTGAGCTCTGATGAGAAAATGTTCGC
CTTTTTTGCTCCGCAGATCCAACAAATGGAGCAGGTTGTTACTGAGCTGG
CAGAGAAAAGTCAGGCTATTCGCGACAACCGGCAGAAACTGAGCCTTATT
CAGACGCAGCTTGCACAACTAATGGTACCTGCCACAGAAAACCATACTGC
CAGATAGATGCATAATGCAGTTCCGTACACCTGTGGTTGAATAACAGCAG
ACTCTTTCATGATTATCTGGATTGAAAGAATGTCTGCTGCTTATTATCGG
AAATATAACTTACTTAACACATGAGGCATAAATCAGTGAGATTATTGTTA
AGGGGCGTTGTTGGAATGATAATGGTCTGGTTGGTTATTTATTTCGGAAT
GGAATTACTTTTTATCTCCGATCGGGTTCATCCGGTATTAAGAATAATTA
4221887 TTGTCCTGGGAATGTTATTCCTGATGATAATGATTGCGGGCGGTATGTGT terminator?
CGTTCTCTTCTGTCACGAAAGAAAACATCATGACGTTCCTGTTTACGCCG
CTACATTTGTGGTGGCTTTATGATTATTCAGAATAATGAACTCACCTGTC **no RBS ?**
GCGGCATGTTCAGCGACCAGCGGCAGACAGAAATATCGCTTTTACTCAAT
TATTATGAAAATTAATCTTCCGTTTATACACTGGCCGCGGATTTGCAATG
ATAAGCGTGTCGTGTTATCTGTGGCTGCCTTTACCCTTTCTAATCTCCTT
GTTTCCTCCGGCGTGCATCTGATCTACCGGGTGTTATTCTTTATACTGGT
ACTTCTTATTATTAAAGCAATGAAATCGATTACGTTGCGTTTGATTCTTG
CCTTCCCGTTCACATTACTGACTGCTGCAGATATCAGTATCAGCCTGTAT *orf5*
TCATGGTGTACATTTGGCACAACATTTAATGATGGTTTTGCAATCAGTAT
4221387 CCTTCAGACAGATCCTGATGAAGTAATAAGAATGTTCAGGATGTATGTTG
TTTATGTTATCGCATTTATTATCCTCTTTTTATTGTTCTTCTGTTCAGCA
ATAAATAAAACGTCCTCATTTCCATCGGAAAATGTCACGGTAATTACATT
TTTATTGTTAATTACAGTAACGTTATATTCTTCCTTCCAGTTTGCGTTGA
AGAAACAGTATCAAATAAATGAAGTTGATCCTTACATAGTCGCTTCCCGG
TTTGCCACATATACCCCTTTCTTTAATCTGAACTATTTTGCACTGGCCGC

	AAAGGAGCATCAGAGATTAATGACGATTGCTGATACCATACCTCACTACG	
	ATCTGGTGATTACGGATAACAACACTGACGTATTTGTTCTGGTGATTGGT	
	GAATCTGCAAGGACTGATAACATGTCCATTTACGGGTATTCGCGCCCAAC	
	CACACCAGAGCTTCAGAAACAGAAATCACGACTGAAACTGTTCACTCAGG	
4220887	CAATCAGCGGGGCACCTTATACCGCCCTTGCGGTACCGCTGGCATTATCT	
	GCTGATACCGTGCTGCATCATGATGTTCGCCGTTACCCCGATAATATTAT	
	CAACATGGCGAATCAGGCGGGGTTCGATACCTGGTGGCTCAGTTCGCAGT	*orf5*
	CCGCTTTCCGGCAGAACGGAACGGCTGTGGCCAGTATCGCCATGCGGGCC	
	AGAAACAGAATTTATGTCAGAGGCTATGATGAGTTACTTCTCCCACACCT	
	GGCTGAAGCACTGAACAGTAATCCGGGGAGCAGGAAACTGATTGTTCTGC	
	ATCTGACGGGCAGCCATGAGCCAGTCTGCAGTAACTGGCCCAGAGATAAA	
	GCCGTGTTTAAACCGCTGGATACAGAAGAAGTCTGCTACGATAATTCCAT	
	TCATTATACCGACAGTTTGTTGGGACAGGTTTTCGCTATGCTGGAAACGC	
	GTCGGGCATCCGTCATGTATTTTTCAGATCACGGTCTGGAGTATGACCCG	
4220387	ACAAAAGAACATGCCTATTTTCATGGTGGAATAAAACCCAGCCAGCAGGC	
	TTATCATGTTCCGATGTTTATCTGGTACAGCCCGACACTGGGAGAGCATG	
	TCGATCGCCAGACGGTAAACAGCGTTTTTTCCACTGCGTATAATGATTAT	
	CTGATTAATGCATGGATGGGCGTAACCAGACCGGCTCAACCGAAAACACC	
	GG**AGGAGG**TGATTACCCGCTGGCAGGGAAAATCAGATGTTTTTGATGCAA	
	ATCATAACGTGTTTGACTATAACGTATTGCGAAAGAATTTTAATGAGCTC	
	ATGGAATAATTCGTTAAAGTAATTTACTACGCTATTTGTTCAAAGGAAAC	terminator?
	TTTCAGAAAATAACCCCAGGGCGATAACCTGGGGAAGGGGAACTGTGTTA	
	TTACTGGTGGATAAATTATTACTTGTTTGAAAGATACACTAATGCAATAT	
	CTACTGATGCTGGATGCTTGAGTGTTTCATTCCTGCTGCTGAAAACGGCA	
4219887	ATATCCAGGGCCAGACATATTCACCGTTAATATTTCTGCATTTCTGTACA	

Numbers refer to NCBI NC_017633.1, *E. coli* ETEC H10407 complete genome sequence entry (as of March 2013) (Crossman et al., 2010). Start and Stop codons are underlined, putative ribosome binding sites (RBSs) are in bold, putative terminators highlighted in purple, putative promoter highlighted in red. The region between *leoC* and *leoB* was confirmed by re-sequencing of our H10407 strain since it clearly shows the end and beginning of two genes.

Figure S2. Top: LeoA-3xFLAG immunofluorescence shows foci predominantly at the poles and in the cell centre. Approximately 250 cells were measured. Foci were measured and placed into 5 bins indicating their localisation within a cell length expressed as a percentage of cell length: for example the 40-50/50-60 bin shows number of foci that were within a band at mid cell (between 40-60 % of cell length). Bottom: field view showing selected and measured cells, demonstrating random selection.

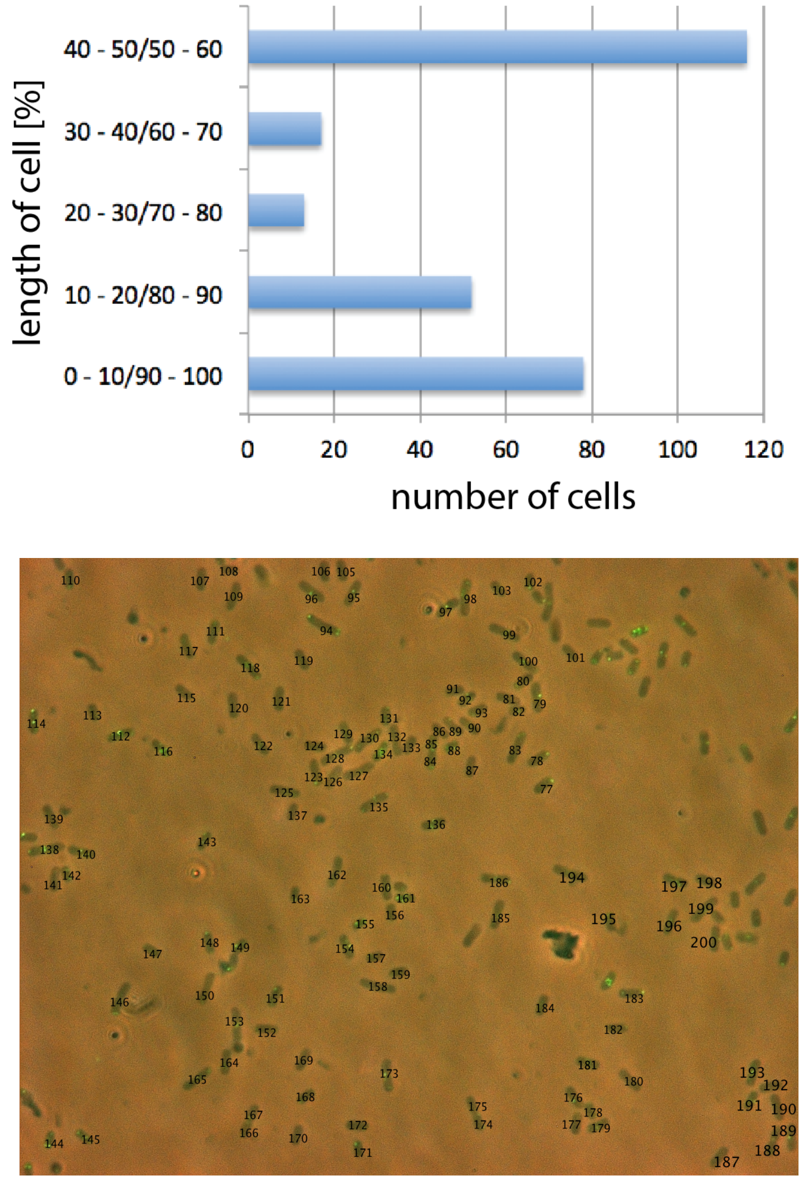

Figure S3. LeoA-His$_6$ does not bind or hydrolyse GTP. A: Equilibrium dialysis. Performed according to Supplementary methods using [α-32P] GTP and dialysis cassettes. B: Radioactive filter binding assay with FtsZ as positive control. C: Colourimetric GTPase assay using malachite green (PiColorlock, Innova Biosciences).

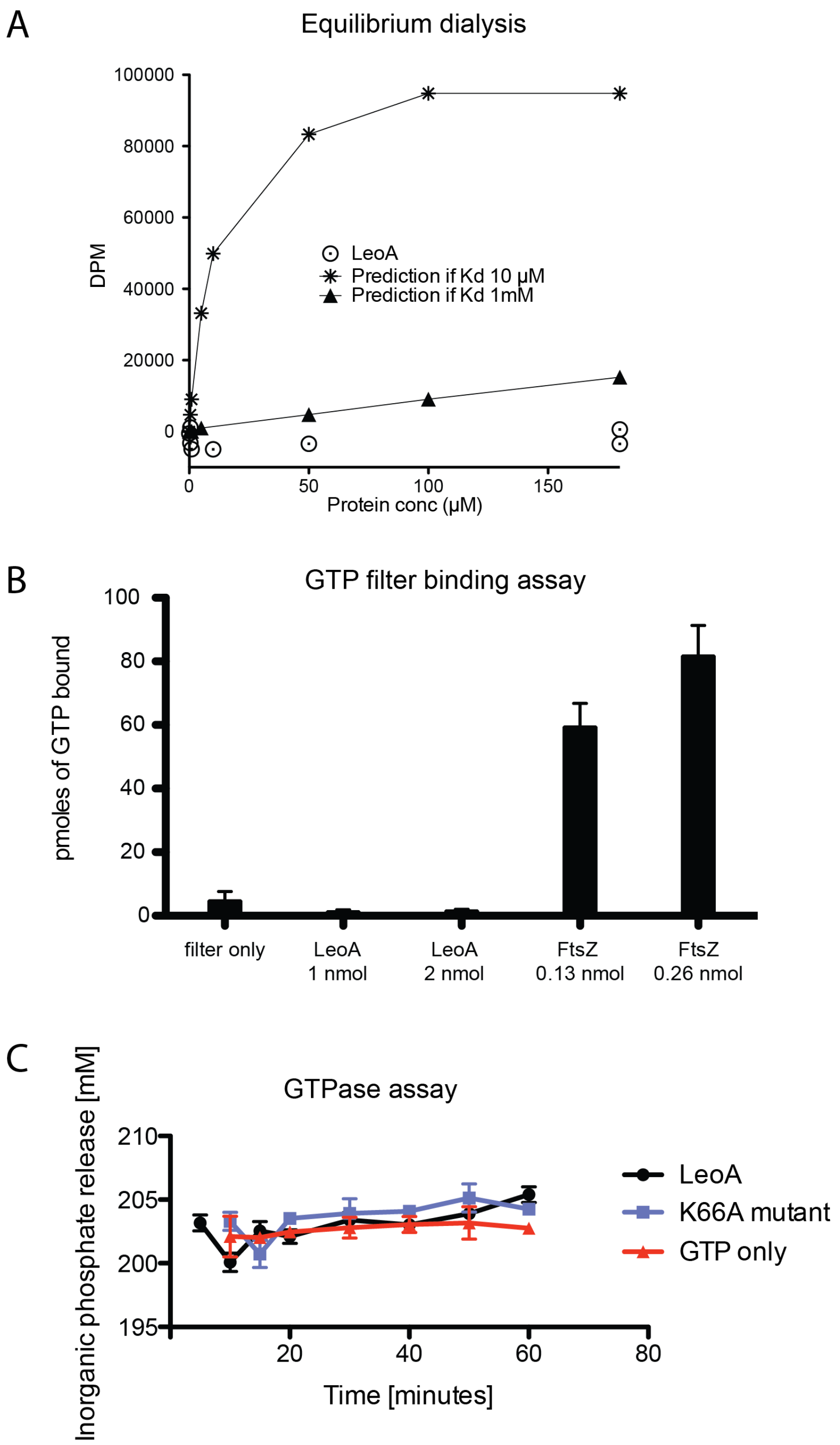

Figure S4. Deletion of *leoA* has no obvious effects on growth or cell morphology in ETEC. A: H10407 and H10407Δ*leoA* were grown in LB at 37°C. OD_{600} were measured every 40 min. and plotted. B: DIC microscopy of both stationary and exponentially growing strains. Size bar = 5 μm

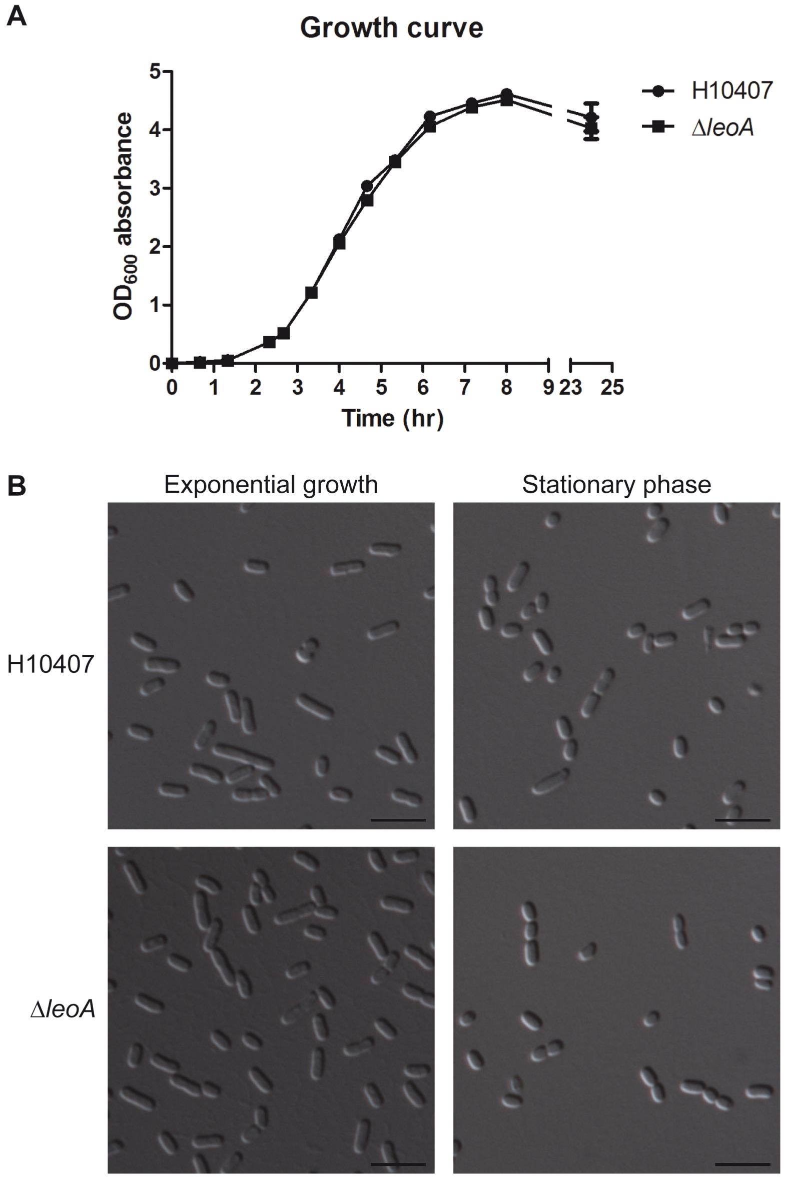

Figure S5. LeoA periplasmic localisation is not affected by the detection method or deletion of *leoB*. Top: same experiment as in Figure 4A, but using a 3xFLAG-tagged version of LeoA and an α-FLAG antibody. (H10407 and H10407LeoA-3xFLAG grown in parallel; protein fractionation as described in methods. Protein loaded on gel, blotted onto membrane, probed first with α-FLAG antibodies (top blot), stripped and subsequently probed with α-LeoA antibodies (lower blot). Bottom: H10407 and H10407Δ*leoB* knockout mutant were grown in parallel; protein fractionation as described in methods.

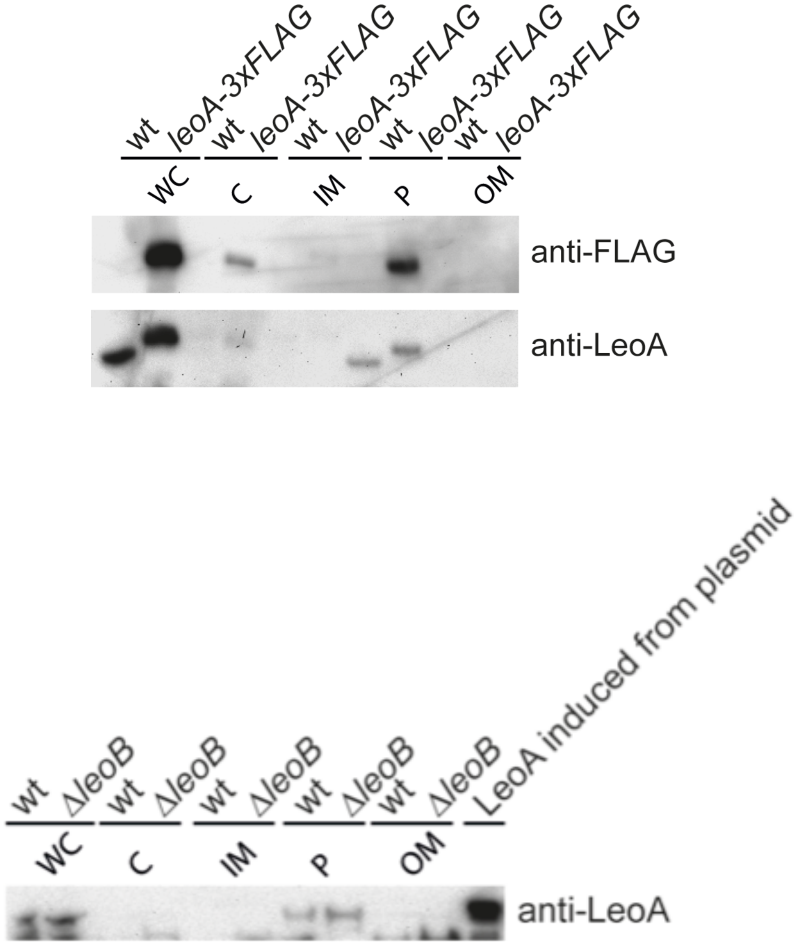

Figure S6. Western blots and quantification showing Tat-GFP and OmpA levels in *leo* deletion strains and vesicle fractions derived from these. This figure provides the original blots and quantification data used for the quantification as shown in Figure 4D.

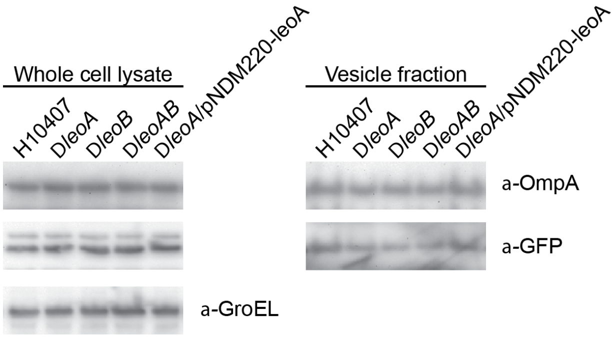

Quantification experiment 1:

Whole cell lysate (GroEL loading control)

	Relative levels GFP	OmpA
H10407	100	100
leoA	104	116
leoB	118	115
leoAB	124	103
leoA/pNDM220-leoA	131	84

Enriched vesicles (No loading control)

	Relative levels GFP	OmpA
H10407	100	100
leoA	66	56
leoB	59	57
leoAB	68	57
leoA/pNDM220-leoA	136	111

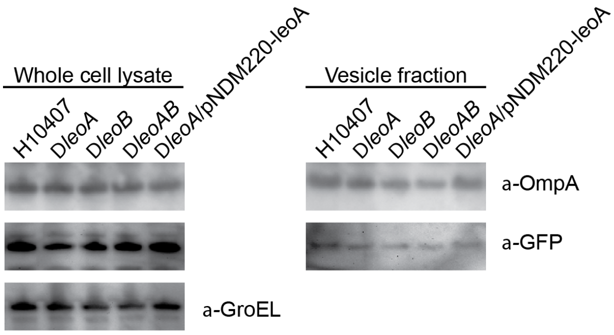

Qunatification experiment 2:

Whole cell lysate (GroEL loading control)

	Relative levels GFP	OmpA
H10407	100	100
leoA	97	104
leoB	109	123
leoAB	144	124
leoA/pNDM220-leoA	111	87

Enriched vesicles (No loading control)

	Relative levels GFP	OmpA
H10407	100	100
leoA	48	51
leoB	50	45
leoAB	56	34
leoA/pNDM220-leoA	97	93

Table S1: List of strains and plasmids used in this study.

Strains/plasmids	Genotype	Source/reference
Strains		
Top10	F- *mcrA* Δ(*mrr-hsd*RMS-*mcrBC*) φ80*lac*ZΔM15 Δ*lac*X74 *nup*G *rec*A1 *ara*D139 Δ(*ara-leu*)7697 *gal*E15 *gal*K16 *rps*L(Str^R) *end*A1 λ$^-$	Invitrogen
BL21-AI	F$^-$ *omp*T *hsd*S_B (r_B^- m_B^-) *gal dcm ara*B::T7RNAP-*tet*A	Invitrogen
H10407	O78:H11:K80	(Evans, Jr. and Evans 322-28)
AB109	H10407 *leoA* 3xFLAG	This work
AB110	H10407Δ*leoA*	This work
AB111	H10407Δ*leoB*	This work
AB112	H10407Δ*leoAB*	This work
AB113	H10407/pAB107	This work
AB114	H10407Δ*leoA* /pAB107	This work
AB115	H10407Δ*leoB* /pAB107	This work
AB116	H10407Δ*leoAB* /pAB107	This work
AB117	H10407Δ*leoA* /pAB107/pAB108	This work
Plasmids		
pKD46	λ-Red expression vector, *Rep*^{ts}, *Amp*^{R}	(Datsenko and Wanner 6640-45)
pKD3	Template plasmid containing an FRT-flanked chloramphenicol resistance gene	(Datsenko and Wanner 6640-45)
pCP20	FLP recombinase expression, *Rep*^{ts}, *Amp*^{R}, *Cml*^{R}	(Datsenko and Wanner 6640-45)
pSUB11	Plasmid with a 24 nt 3xFLAG sequence and FRT sites flanking a kanamycin resistance cassette	(Uzzau et al. 15264-69)
pAB107	*torA-gfp* in pXG-10	This work
pAB108	*IPTG indicible leoA* in pNDM220	This work
pXG-10	pSC101 derivative, constitutive P_{LtetO} promoter	(Urban and Vogel 1018-37)
pNDM220	Mini-R1, *bla*, *LacIq*, PA1/O4/O3	(Gotfredsen and Gerdes 1065-76)
pHLLeoA	LeoA in phis17 with N-terminal hexahistidine tag	This work
pKM100	*leoC-leoB -leoA* in phis17	This work
phis17	Bruno Miroux, MRC-LMB personal communication	

Table S2: List of oligonucleotides used in this study.

Primer name	Sequence
JMJ91	ATGGAACAATTCAAACAGTTCAGTTATTGAAAAACAGGCTGCGATTAACTCGTGTAGGCTGGAG CTGCTTCG
JMJ92	CTATCTGGCAGTATGGTTTTCTGTGGCAGGTACCATTAGTTGTGCAAGCTCATATGAATATCCT CCTTAGTTCC
JMJ101	CTCTCGACAGCGTTGCCGATG
JMJ102	CTCACTGATTTATGCCTCATG
JMJ134	GAAACTGAGCCTTATTCAGACGCAGCTTGCACAACTAATGGTACCTGCCACAGAAAACCATACT GCCAGAGACTACAAAGATGACGACGA
JMJ135	GTGTTAAGTAAGTTATATTTCCGATAATAAGCAGCAGACATTCTTTCAATCCAGATAATCATGA AAGAGTCATATGAATATCCTCCTTAG
JMJ205	GCCTGACGTCGGCAAAAAGAGTGTTGACTTGTGAGCGGATAACAATGATACTTAGATTCATGGA ACAATTCAAACAGTTCAGTA
JMJ206	CCCCGGATCCCTATCTGGCAGTATGGTTTTCTGTG
JMJ238	atgagtgatacatggtcttctgtagaggagaatccggagcgggtattcagggatactcacgtgt aggctggagctgcttcg
JMJ239	AAGGCATGCCCTCCAGTTGTTTTTTTAATGTTGTGATTTGACTGATTTGTGTTTCGAAAACATA TGAATATCCTCCTTAGTTCC
JMJ240	TAATATTTCTGTCTATTATTGTTG
JMJ246	CGCTAATGAAAGTCAGTCTCGTTTGCAAATTT
JMJ247	AACGCCCCTTAACAATAATCTCAC
JMJ256	tagcATGCAT GCAAATGAATGCGTCTGACACC
JMJ257	tagcGCTAGC CGCCGCTTGCGCCGCAGTCGC
JVO-155	CCGTATGTAGCATCACCTTC
pZE-CAT	TGGGATATATCAACGGTGGT
KM223	GGAATTCCATATGGATGTGAACAGGGCACAATG
KM225	GCGAGATCTCTATCTGGCAGTATGGTTTTC

Supporting Information References

Crossman, L. C., Chaudhuri, R. R., Beatson, S. A., Wells, T. J., Desvaux, M., Cunningham, A. F., Petty, N. K., Mahon, V., Brinkley, C., Hobman, J. L., Savarino, S. J., Turner, S. M., Pallen, M. J., Penn, C. W., Parkhill, J., Turner, A. K., Johnson, T. J., Thomson, N. R., Smith, S. G., and Henderson, I. R. (2010). A commensal gone bad: complete genome sequence of the prototypical enterotoxigenic *Escherichia coli* strain H10407. J. Bacteriol. 192, 5822-5831.

Datsenko KA, Wanner BL: One-step inactivation of chromosomal genes in Escherichia coli K-12 using PCR products. Proc Natl Acad Sci U S A 2000, 97:6640-6645.

Evans, D. J., Jr. and D. G. Evans. "Three characteristics associated with enterotoxigenic *Escherichia coli* isolated from man." Infect.Immun. 8.3 (1973): 322-28.

Gotfredsen, M. and Gerdes, K. (1998) Mol. Microbiol. 29, 1065-1076

Urban, J. H. and J. Vogel. "Translational control and target recognition by *Escherichia coli* small RNAs in vivo" Nucleic Acids Res. 35.3 (2007): 1018-37.

Uzzau S, Figueroa-Bossi N, Rubino S, Bossi L: Epitope tagging of chromosomal genes in *Salmonella*. Proc Natl Acad Sci U S A 2001, 98:15264-15269.
